# Hookah use prevalence, predictors, and perceptions among Canadian youth: findings from the 2012/2013 Youth Smoking Survey

**DOI:** 10.1007/s10552-015-0556-x

**Published:** 2015-03-18

**Authors:** Leia M. Minaker, Alanna Shuh, Robin J. Burkhalter, Steve R. Manske

**Affiliations:** Propel Centre for Population Health Impact, University of Waterloo, 200 University Ave W, Waterloo, ON N2L3G1 Canada

**Keywords:** Hookah smoking, Water-pipe smoking, Adolescent, Canada, Youth Smoking Survey, Tobacco control

## Abstract

**Purpose:**

Few national surveys currently assess hookah smoking among youth. This study describes the prevalence, patterns of use, and perceptions about hookah in a nationally representative survey of Canadian grades 9–12 students.

**Methods:**

The Youth Smoking Survey 2012/2013 was administered to 27,404 Canadian grades 9–12 students attending schools in nine Canadian provinces representing 96 % of Canadian population. Relevant dichotomous outcomes included ever use, use in the last 30 days, and the belief that hookah use is less harmful than cigarette smoking. Covariates included smoking status, sex, grade, province of residence, race/ethnicity, and amount of weekly spending money. Logistic regression models were used to examine: covariates related to the odds of ever and last-30-day hookah use; covariates related to perceptions about the harms of hookah smoking; the extent to which perceptions were associated with odds of hookah use; and whether survey year (2010/2011 or 2012/2013) was associated with hookah use, and marginal effects were calculated.

**Results:**

In Canada, 5.4 % of students in grades 9–12 currently use hookah and 14.3 % report ever using hookah. In 2012/2013, students had significantly higher odds of using hookah compared to students in 2010/2011 (OR 1.5, 95 % CI 1.2, 2.1). About half of hookah users (51 %) used flavored hookah. Students who believed that hookah use was less harmful than cigarette smoking had significantly higher odds of current hookah use (OR 2.6, 95 % CI 1.9, 3.5), as did students who reported higher amounts of weekly spending money. Current smokers had an 18 % higher predicted probability of currently using hookah compared to non-smokers.

**Conclusions:**

Hookah use among youth is of growing concern in Canada. Findings can be used to inform policy development related to youth hookah smoking.

## Introduction

Globally, tobacco use continues as the leading cause of preventable death [1]. Although the North American prevalence of daily cigarette smoking has decreased since 1980 [2], increases in the prevalence of alternate tobacco product consumption may counteract public health gains resulting from declining cigarette consumption [3, 4]. One notable alternate tobacco product is shisha tobacco (also called narghile, arghile, and hubble-bubble), which is traditionally smoked with a hookah (i.e., water-pipe), a traditional Middle Eastern pipe [5]. Although hookah is an ancient form of tobacco smoking, it is gaining popularity worldwide, especially among youth and young adults [6–8]. Globally, about one billion people are familiar with hookah and approximately 100 million people use hookah on a daily basis [9]. The introduction of manufactured flavors has contributed to the rapid increase in hookah use in western countries [7, 10, 11]. Flavored hookah tastes smooth and sweet, making it attractive to users, especially youth, young adults, and beginner smokers [4, 10–12]. The novel flavors and social experience of using hookah tobacco may diminish users’ perception that hookah smoking is dangerous and may allow continuous smoking for up to 2 hours [13].

The common misperception that hookah is less risky than cigarette smoking is another factor that may contribute to the increasing prevalence of hookah use [7]. For example, many people mistakenly believe that the water filters the smoke as it passes through the water [12, 14]. Although toxicant exposure associated with hookah smoking varies by hookah components and by hookah user, many of the toxic and carcinogenic compounds found in mainstream cigarette smoke are also found in hookah smoke and may even exceed the amounts found in mainstream cigarette smoke [15]. In addition to the novel flavors and common misperceptions around hookah use, many young people view hookah as an affordable, accessible, socially acceptable way to socialize with friends [11, 13]. In many jurisdictions, hookah cafés have a lower age restriction and are less costly than bars where alcohol is served, making hookah an accessible pastime for youth and young adults [13]. Currently, few national surveys address hookah smoking [6]. This is an important gap in national tobacco use surveillance systems, given that longitudinal data indicate that hookah smoking increases user susceptibility to begin cigarette smoking [11, 16, 17]. The high nicotine content in shisha may also make it harder for concurrent cigarette and hookah users to quit smoking [18]. Finally, hookah use causes nicotine dependence and disease [11, 19] and is associated with several types of cancer, respiratory disease, poor pregnancy outcomes (including low birth weight), cardiovascular disease, and periodontal disease [19, 14].

The aim of the current paper is to examine the prevalence of hookah smoking and socio-demographic factors associated with hookah smoking in a nationally generalizable sample of Canadian grades 9–12 students. A secondary objective is to examine students’ perceptions of harm of hookah smoking. We hypothesize that students who perceive hookah smoking to be less harmful than cigarette smoking will have higher odds of using hookah compared to students who perceive hookah smoking to be at least as harmful as cigarette smoking.

## Methods

### Study design

The Youth Smoking Survey (YSS) is a biennial, nationally generalizable school-based, paper-and-pencil survey that measures determinants of tobacco use among youth [20]. The target population was students in grades six through 12 (aged 11–18) at public and private schools (*n* = 450) in nine provinces. Those residing in the province of Manitoba, or territories of Yukon, Nunavut, and Northwest Territories and those living in institutions or on First Nations reserves were excluded (representing about 4 % of the Canadian population) from the 2012/2013 cycle. Surveys were pilot tested to assess the logic and student understanding of the questions. Approximately 73 % of respondents participated with passive parental permission, and 27 % participated with active parental permission. The YSS survey was administered during class time, and participants were not remunerated. Survey development, design, weights, response rates, and data collection protocol for the 2008 YSS have been published [20]. Overall, the school response rate (the percent of schools that participated in the study once approached) was 64 % (range 38 % in Ontario to 96 % in Newfoundland). The overall student response rate (the percent of eligible students within participating schools) was 72 %. The 2012/2013 YSS was administered to 47,203 youths in grades six through 12 attending schools (in Quebec, secondary school ends at grade 11). Given the low prevalence of hookah use among grades 6–8 students (2.0 % had ever tried hookah; 0.9 % reported using hookah in the last 30 days, and 0.3 % had reported using flavored hookah in the last 30 days), this study restricts analyses to grades 9–12 students (*n* = 27,404). Data from the 2012/2013 cycle were collected between November 2012 and June 2013. Data from the 2010/2011 YSS were also used to examine hookah use over time. Recruitment, participation, data entry, and cleaning procedures from the 2010/2011 cycle were identical to those in the 2012/2013 cycle. In 2010/2011, however, data collection excluded youth residing in New Brunswick rather than Manitoba. Data were analyzed in 2014. This study was approved by the University of Waterloo Human Research Ethics Committee, the Health Canada Research Ethics Board, and appropriate School Board and Public Health Ethics committees.

### Measures and data sources

Relevant, dichotomous outcomes from the 2012/2013 YSS dataset included ever use and last 30 day use of hookah, and students’ reporting that they believe using hookah is less harmful than smoking cigarettes. Students were asked, “Have you ever tried any of the following?,” with a variety of response options including “Using a water-pipe (hookah) to smoke shisha (herbal or tobacco),” and “I have not tried any of these things.” Ever users of hookah were defined as those who indicated they had tried the water-pipe (hookah) option. Students were also asked, “In the last 30 days, did you use any of the following?” Response options included a variety of tobacco products, including “a water-pipe (hookah) to smoke shisha (herbal or tobacco),” and “I have not used any of these things in the last 30 days.” Last-30-day hookah users were defined as those who indicated they used a water-pipe (hookah) in the last 30 days. Last-30-day use is a commonly used standard of current use, originating with the Centers for Disease Control. Finally, students were asked, “Do you believe that using a water-pipe (hookah) to smoke shisha (herbal or tobacco) is:” with response options, “More harmful than smoking cigarettes?;” “Less harmful than smoking cigarettes?;” and “Neither more harmful nor less harmful than smoking cigarettes?” Those who responded “Less harmful than smoking cigarettes” were defined as perceiving hookah to be less harmful than cigarettes.

 Independent variables included the respondent’s sex, grade [9–12], province (where the four Atlantic provinces were grouped together based on their cultural similarity and the relatively small n), self-reported race/ethnicity, (white, black, Asian, Aboriginal, Latin American, or “other”), cigarette smoking status, and the amount of weekly spending money (in Canadian dollars) received. Respondents who had smoked 100 or more cigarettes in their lifetime and smoked cigarettes in the past 30 days were considered current smokers and were compared to all others: Former smokers were those who reported smoking 100 or more cigarettes in their lifetime but did not smoke in the last 30 days; non-smokers were those who reported smoking fewer than 100 cigarettes in their lifetime. Weekly spending money was categorized as none, $1–$20, $21–$100, and more than $100.

Differences in ever and last-30-day use of hookah between the 2010/2011 and the 2012/2013 YSS cycles were also assessed. In 2010/2011, ever use and last-30-day use were assessed via the same question format with a slight difference in wording. Ever users of hookah were those who reported ever trying “a water-pipe to smoke tobacco (also known as hookah, shisha, narghile, hubble-bubble, or gouza).” Last-30-day hookah users responded yes to the question, “In the last 30 days, did you use a “water-pipe to smoke tobacco (also known as a hookah, shisha, narghile, hubble bubble, or gouza)?”

### Statistical analysis

Survey weights were used to adjust for sample selection (school and class levels), non-response (school, class, and student levels), and post-stratification of the sample population relative to grade and sex distribution in the total population. Bootstrap weights were used for all regression analyses so that the variances take account of the sample design.

The first objective was to examine the prevalence and correlates of hookah smoking. Descriptive statistics were used to show the prevalence of use of plain and flavored hookah by sex, grade, geographic region, self-reported race/ethnicity, and weekly spending money. Proportions were calculated as percentages to show flavored hookah as a percent of overall hookah use and to show the percent of students who reported believing that hookah use is less harmful than smoking cigarettes. To examine correlates of hookah use among respondents in grades 9–12 with complete data for the variables of interest, two logistic regression models were created to examine independent variables related to the odds of ever and current (last-30-day) use of hookah.

The second objective was to examine students’ perceptions of the harm of hookah smoking and to test whether these perceptions were significantly associated with hookah use. To examine students’ perceptions of hookah smoking, a logistic regression model was created to examine independent variables related to the odds of a student believing hookah is less harmful than smoking cigarettes. Independent variables included sex, grade, geographic region, self-reported race/ethnicity, cigarette smoking status, and weekly spending money. Finally, to examine whether beliefs about hookah use were associated with hookah use, a fourth logistic regression model was created to examine whether beliefs about harms associated with hookah use were associated with odds of current hookah use among all respondents. Covariates included sex, grade, geographic region, self-reported race/ethnicity, cigarette smoking status, and weekly spending money. Finally, a fifth logistic regression model was fitted with all covariates to examine whether survey year (2010/2011 or 2012/2013) was significantly associated with last-30-day hookah use.

Full models initially consisted of main exposure, outcome, and covariates. Assumptions of logistic regressions (e.g., sufficient sample size for single cell counts) were checked, and Akaike’s information criterion (AIC) goodness-of-fit tests were used to check model fit. The final models showed the measure of association between an independent variable of interest and outcome. Logistic regressions were conducted by using PROC SURVEYLOGISTIC in SAS 9.3 (SAS Institute Inc, Cary, North Carolina). To aid interpretation of increases or decreases in probability of hookah use, conditional marginal effects were calculated using the LSMEANS (least squares means) statement in the PROC SURVEYLOGISTIC procedure in SAS, using the “OM” (observed margins) along with the “ILINK” (inverse link) option.

## Results

In Canada in 2012/2013, 37.6 % of grades 9–12 students thought hookah use was less harmful than smoking cigarettes, 14.3 % reported ever using a hookah, 5.4 % reported using a hookah in the last 30 days, and 2.7 % reported using a flavored hookah in the last 30 days (see Table 1). Several provincial hookah use estimates are subject to moderate sampling variability as noted in Table 1. Hookah ever use ranged from 11.7 % in British Columbia to 17.7 % in Alberta, while last-30-day hookah use ranged from 4.1 % in British Columbia to 7.1 % in Alberta. Whereas 10.8 % of youth who never smoked had ever tried a hookah, 55.8 % of current smokers had ever tried a hookah. Almost half (47.9 %) of current smokers believed hookah use was less harmful than smoking cigarettes, compared to 36.7 % of never smokers who believed hookah use was less harmful than smoking cigarettes.

**Table 1:** Weighted prevalence of hookah ever use and last-30-day use (total and flavored), as well as perceptions about harm: grades 9–12, Canada, 2012/2013 YSS

Characteristics of survey population	*n* (%)^a^	Ever use of hookah (%)	Last-30-day use of hookah (%)	Use of flavored hookah (%)	Use of flavored tobacco among hookah users (%)	Believing hookah use is less harmful than smoking cigarettes (%)
Canada	27,404 (100)	14.3	5.4	2.7	51.4	37.6
Gender						
Female	13,880 (50.6)	12.5	4.0	2.1	54.6	36.7
Male	13,524 (49.4)	16.0	6.6	3.2	49.6	38.5
Grade						
9	7,066 (25.8)	7.3	3.5^b^	1.5	42.9	31.3
10	7,680 (28.0)	11.7	4.8	2.2	48.4	37.5
11	7,114 (26.0)	16.1	5.8	2.9	51.3	38.2
12	5,544 (20.2)	22.7	7.6	4.4	57.7	43.5
Provinces						
Ontario	4,438 (16.2)	14.6	5.3	2.8	53.4	38.0
Atlantic	9,531 (34.8)	12.8	5.2	2.4	47.9	33.4
Quebec	2,701 (9.9)	14.1	5.5^b^	2.3	42.4	31.4
Saskatchewan	3,714 (13.6)	13.5	5.1	2.9	57.5	38.6
Alberta	3,416 (12.5)	17.7	7.1^b^	3.9	55.1	45.9
British Columbia	3,604 (13.2)	11.7^b^	4.1^b^	2.1^b^	52.9	39.0
Ethnicity						
White	19,322 (70.9)	13.5	4.3	2.0	46.5	38.0
Asian	2,692 (9.9)	9.9	3.9	2.5^b^	66.1	35.0
Aboriginal	1,916 (7.0)	19.4	8.5	4.3^b^	52.4	35.5
Black	1,161 (4.3)	13.7	7.2	3.3^b^	47.2	38.4
Latin	433 (1.6)	24.6	11.8	7.8	65.8	41.1
Other	1,721 (6.3)	19.4	10.0^b^	5.5^b^	55.6	37.1
Smoking status						
Current smoker	2,362 (8.6)	55.8	26.9	15.5	59.4	47.9
Former smoker	308 (1.1)	50.4	11.1^b^	5.0^b^	NR	47.7
Non-smoker	24,734 (90.3)	10.8	3.7	1.7	47.4	36.7
Weekly spending money						
No money	4,182 (18.6)	7.9	2.7^b^	0.9^b^	34.1^b^	31.0
$1–20	7,227 (32.2)	12.0	4.2	1.6^b^	39.5	37.6
$21–100	6,894 (30.7)	18.4	6.8	3.4	52.1	42.0
More than $100	4,155 (18.5)	26.1	11.8	7.0	60.3	45.0

Grades 9–12, Canada, 2012/2013 YSS

NR High sampling variability, data are suppressed

*YSS* youth smoking survey, *N* number

^a^Unweighted sample sizes and estimates in this column, all other estimates are weighted

^b^Moderate sampling variability, interpret with caution (marginal estimates have a sample size of 30 or more and high coefficients of variation in the range of 16.5–33.3 %)

As shown in Table 2, males had significantly higher odds of ever using hookah (OR 1.2, 95 % CI 1.0, 1.4) and last-30-day hookah use (OR 1.5, 95 % CI 1.1, 2.0) compared to females. Results from marginal estimates calculations reveal that the predicted probability of currently using hookah was 1.6 % higher among males compared to females. Relative to grade nine students, students in older grades had significantly higher odds of ever using hookah and significantly higher odds of believing hookah is less harmful than cigarette smoking, although last-30-day hookah use did not vary significantly by grade. In terms of current hookah use, marginal estimates with grade nine students as the reference group ranged from 0.7 % higher predicted probability for grade ten students to 1.4 % higher predicted probability for grade 12 students. Provincial rates of hookah use and believing hookah is less harmful than cigarette smoking did not vary significantly with the exception of Alberta, where students had significantly higher odds of ever using hookah (OR 1.4, 95 % CI 1.0, 1.8) and believed hookah is less harmful (OR 1.5, 95 % CI 1.1, 2.0) relative to students in Ontario. Differences in predicted probability of current hookah use ranged from 1.4 % lower for students in British Columbia to 1.6 % higher for students in Alberta compared to students in Ontario. Relative to students who identified as white, students who identified as other had significantly higher odds of ever use (OR 1.9, 95 % CI 1.4, 2.5) and last-30-day use (OR 3.5, 95 % CI 2.1, 5.7). Compared to white students, students who identified as black had significantly higher odds of last-30-day use (OR 1.9, 95 % CI 1.2, 3.0) and students who identified as Latin also had significantly higher odds of last-30-day use (OR 2.8, 95 % CI 2.0, 4.0). Students identifying as white had the lowest predicted probability of currently using hookah. In terms of other self-identified ethnicities, predicted probability was higher for students identifying as Asian (1.1 % higher predicted probability), Aboriginals (1.8 % higher predicted probability), black (2.8 % higher predicted probability), Latin (5.6 % higher predicted probability), and other (7.3 % higher predicted probability). Relative to non-smokers, current smokers had significantly higher odds of ever use (OR 8.0, 95 % CI 6.0, 10.6), last-30-day use (OR 7.6, 95 % CI 5.8, 9.9), and believing that hookah use is less harmful than cigarette smoking (OR 1.5, 95 % CI 1.2, 1.8). Current smokers had 18.4 % higher predicted probability of current hookah use compared to non-smokers. Relative to students with no weekly spending money, students with any amount of weekly spending money had significantly higher odds of ever using hookah, last-30-day use of hookah, and believing hookah is less harmful than cigarette smoking. Differences in the predicted probability of current hookah use ranged from 1.3 % higher for students receiving $1–20 per week to 5.5 % higher for students receiving more than $100 per week compared to students who receive no money per week.

**Table 2:** Logistic regression analysis^b^ of variables related to the odds of hookah use and beliefs about hookah harm

Predictors	Ever use of hookah (Model 1: *n* = 22,084) OR adjusted (95 % CI)	Last-30-day hookah use (Model 2: *n* = 21,904) OR adjusted (95 % CI)	Believing hookah is less harmful than cigarettes (Model 3: *n* = 21,068) OR adjusted (95 % CI)
Gender			
Female (ref)	1.0	1.0	1.0
Male	1.2 (1.0, 1.4)	1.5 (1.1, 2.0)	1.1 (1.0, 1.2)
Grade			
9 (ref)	1.0	1.0	1.0
10	1.5 (1.0, 2.2)	1.2 (0.8, 1.7)	1.3 (1.1, 1.4)
11	2.0 (1.5, 2.6)	1.2 (0.8, 1.8)	1.3 (1.2, 1.5)
12	2.8 (2.2, 3.6)	1.4 (0.9, 2.3)	1.5 (1.3, 1.8)
Provinces			
Ontario (ref)	1.0	1.0	1.0
Atlantic	0.7 (0.6, 0.9)	0.8 (0.7, 1.0)	0.8 (0.6, 1.0)
Quebec	1.2 (0.9, 1.7)	1.2 (0.8, 1.9)	0.8 (0.5, 1.1)
Saskatchewan	0.7 (0.5, 1.0)	0.7 (0.5, 1.0)	1.0(0.7, 1.4)
Alberta	1.4 (1.0, 1.8)	1.4 (0.8, 2.3)	1.5(1.1, 2.1)
British Columbia	0.8 (0.4, 1.4)	0.7 (0.3, 1.4)	1.2 (0.8, 1.7)
Ethnicity			
White (ref)	1.0	1.0	1.0
Other	1.9 (1.4, 2.5)	3.5 (2.1, 5.7)	0.9 (0.7, 1.1)
Black	1.2 (0.8, 1.7)	1.9 (1.2, 3.0)	1.1 (0.8, 1.5)
Latin	2.2 (1.6, 3.2)	2.8 (2.0, 4.0)	1.1 (0.8, 1.5)
Asian	1.0 (0.6, 1.4)	1.4 (0.9, 2.0)	0.9 (0.7, 1.1)
Aboriginal	1.2 (0.9, 1.7)	1.6 (0.9, 2.7)	0.8 (0.6, 1.0)
Smoking status			
Non-smoker^a^ (ref)	1.0	1.0	1.0
Current smoker	8.0 (6.0, 10.6)	7.6 (5.8, 9.9)	1.5 (1.2, 1.8)
Weekly spending money			
No money (ref)	1.0	1.0	1.0
$1–20	1.6 (1.4, 1.9)	1.6 (1.0, 2.6)	1.3 (1.2, 1.5)
$21–100	2.2 (1.8, 2.7)	2.4 (1.5, 3.8)	1.5 (1.3, 1.8)
More than $100	2.8 (2.3, 3.6)	3.7 (2.1, 6.4)	1.6 (1.3, 1.9)

Grades 9–12, Canada, 2012/2013 YSS

*YSS* youth smoking survey, *N* number, *OR* odds ratio, *CI* confidence interval

^a^Non-smokers are defined as never smokers and former smokers

^b^All logistic regressions were conducted using a complete case methods approach, so findings presented here are among all cases with complete data

Students who believed that hookah use was less harmful than cigarette smoking had significantly higher odds of current hookah use in the last 30 days (OR 2.6, 95 % CI 1.9, 3.5), even after controlling for smoking status, gender, grade, geographic region, ethnicity, and weekly spending money, as shown in Table 3.

**Table 3:** Logistic regression analysis of the odds of 30-day hookah use on beliefs about hookah harm

Predictors	Last-30-day hookah use among respondents with complete data (Model 4: *n* = 20,731)^a^ OR adjusted (95 % CI)
Perceptions	
Hookah at least as harmful as cigarettes (ref)	1.0
Hookah less harmful than cigarettes	2.6 (1.9, 3.5)
Gender	
Female (ref)	1.0
Male	1.5 (1.1, 2.0)
Grade	
9 (ref)	1.0
10	1.1 (0.8, 1.6)
11	1.1 (0.7, 1.7)
12	1.3 (0.8, 2.1)
Provinces	
Ontario (ref)	1.0
Atlantic	0.9 (0.7, 1.2)
Quebec	1.3 (0.9, 2.0)
Saskatchewan	0.7 (0.5, 1.0)
Alberta	1.3 (0.8, 2.2)
British Columbia	0.6 (0.3, 1.3)
Ethnicity	
White (ref)	1.0
Other	3.6 (2.1, 6.2)
Black	1.9 (1.1, 3.3)
Latin	2.9 (2.0, 4.2)
Asian	1.4 (0.9, 2.1)
Aboriginal	1.6 (0.9, 2.8)
Smoking status	
Non-smoker (ref)^a^	1.0
Current smoker	7.4 (5.5, 9.8)
Weekly spending money	
No money (ref)	1.0
$1–20	1.7 (1.0, 2.7)
$21–100	2.3 (1.4, 3.7)
More than $100	3.6 (2.0, 6.3)

Grades 9–12, Canada, 2012/2013 YSS

*YSS* youth smoking survey, *N* number, *OR* odds ratio, *CI* confidence interval

^a^Non-smokers are defined as never smokers and former smokers

Finally, compared to 2010/2011, in 2012/2013, Canadian grades 9–12 students had significantly higher odds of using hookah in the last 30 days (OR 1.5, 95 % CI 1.2, 2.1). In 2010/2011, 4.0 % of grades 9–12 students reported smoking hookah in the last 30 days, and in 2012/2013, 5.4 % of grades 9–12 students reported smoking hookah in the last 30 days. After accounting for sex, grade, provincial distribution, weekly spending money, and self-reported ethnicity, using marginal estimates, the predicted probability of currently using hookah was 1.4 % greater in 2012/2013 than it was in 2010/2011.

## Discussion

In Canada, one in twenty students in grades 9–12 reports currently using hookah. Hookah use among grades 9–12 students increased significantly since 2010. Just over half (51 %) of youth hookah users reported using flavored hookah in the past 30 days, indicating that flavored hookah is a popular choice among those using hookah. Current smokers have an 18.5 % higher predicted probability of currently using hookah than non-smokers. Finally, students with more spending money have significantly higher odds of hookah use compared to those with no spending money. Each of these findings is described in more detail below.

First, hookah use is increasing in popularity among youth in many countries [8, 12, 21], a finding which is supported by the current study. In 2012/2013, over 5 % of Canadian grades 9–12 students used hookah in the last 30 days, which is just less than half of the roughly 12 % of Canadian grades 9–12 students who smoked cigarettes in the last 30 days (YSS 2012/2013, unpublished data). Importantly, these data show a continuing and troubling trend of increased hookah use among Canadian youth since 2006. Between 2006 and 2010, for example, the proportion of youth who ever used hookah increased significantly [22]. In 2011, roughly 3 % of students aged 13–17 in the USA smoked hookah in the last 30 days [23], which is slightly lower than the prevalence of youth hookah smoking found in our study. Among grade 12 students, however, nationally representative US data from 2010 to 2012 showed that 18 % reported using hookah in the past year [24]. We did not have data on hookah use in the past year, but found a comparably high percentage of grade 12 students reported ever using hookah (23 %).

Second, about half (51 %) of youth hookah users reported using flavored varieties of hookah in the past 30 days. The introduction of flavored hookah tobacco is thought to be one of the major contributors to the increasing prevalence of hookah use among youth [10–12]. Given the current global policy developments related to flavored tobacco [25–27], this paper provides policy-relevant evidence to federal and state or provincial decision-makers that flavored hookah tobacco is a very popular choice among youth hookah users.

Third, even after adjusting for smoking status, the odds of currently smoking hookah were 2.6 times higher than for those who reported that hookah is less harmful than cigarettes compared to youth who report that hookah is at least as harmful as cigarettes. Mistaken perceptions about hookah use (i.e., that it is less harmful than cigarette smoking) may be fuelled by the flavors commonly found in hookah tobacco, since it makes the smoking experience less harsh [13, 28]. These findings imply a role for public health education campaigns aimed at correcting misperceptions of harms associated with hookah smoking. Current smokers had significantly higher odds of reporting that hookah use is less harmful than cigarette smoking and had 18 % higher predicted probability of currently using hookah compared to non-smokers. This is consistent with data from the Monitoring the Future study, which found that smokers had significantly increased odds of using hookah in the past year relative to never smokers [24]. Certainly, the co-use of cigarettes and hookah and the simultaneous increase in prevalence of hookah use and decrease in prevalence of cigarette use over time suggest that governments should consider how to limit non-traditional tobacco use among youth, despite challenges associated with regulating non-cigarette tobacco products [29].

Finally, ours is the first Canadian study to explore associations between weekly spending money and hookah use. We found, similar to nationally representative data from US high school seniors [24], that youth with higher weekly spending money had significantly higher odds of ever or currently using hookah. The YSS does not contain information on where youth access hookah. If youth frequent hookah bars, the cost of patronizing hookah bars may help to explain the association between weekly spending money and hookah use.

Limitations of the current study include the cross-sectional nature of the survey, which renders the question of whether hookah use is a gateway to cigarette smoking unanswerable. In addition, no data were collected from Canada’s three territories or from the province of Manitoba in the YSS 2012/2013 wave, those living on Aboriginal reserves, or those who do not attend traditional schools. On the other hand, non-included populations represent only approximately 4 % of the Canadian population. An additional limitation was the complete case methods approach, which is commonly used in public health research but may provide biased estimates when there is a high prevalence of missing data. However, these methods are in keeping with other studies using YSS data (e.g., [18]). Strengths of the study include use of provincially generalizable samples of Canadian grades 9–12 students from nine provinces, reliable and valid survey instruments, and the examination of hookah use prevalence over time.

Given that many non-smokers may be introduced to tobacco through products other than cigarettes [7], tighter regulation of other tobacco products that are marketed to and popular among youth may reduce overall tobacco use rates. However, challenges associated with regulating non-cigarette tobacco products relative to cigarettes have caused legislation and regulation of other tobacco products to lag behind the regulation of cigarettes [30]. As of 2010 in the USA, for example, 73 of the 100 largest cities had clean air regulations disallowing cigarette smoking in bars. Of these, only four cities (6 %) had comprehensive legislation that did not appear to exempt hookah tobacco smoking [31]. In Canada, there is a small but growing number of municipalities that have prohibited hookah smoking in restaurants, bars, cafes, patios, and even outdoors on municipal property [32]. To date, two Canadian provinces have prohibited hookah smoking in public places, but in some cases, cigar and hookah bars are exempt from legislation [32].

Few national tobacco surveillance systems address hookah use [6]. Results suggest that tobacco use surveillance systems should include alternate tobacco product use, since excluding certain types of other tobacco products underestimates prevalence estimates of tobacco use [33]. In addition, the question of where and how youth access hookah is an important topic for future research.
